# Effects of Partial Replacement of Corn with Glycerin on Ruminal Fermentation in a Dual-Flow Continuous Culture System

**DOI:** 10.1371/journal.pone.0143201

**Published:** 2015-11-23

**Authors:** Pedro Del Bianco Benedeti, Lorrayny Galoro da Silva, Eduardo Marostegan de Paula, Teshome Shenkoru, Marcos Inácio Marcondes, Hugo Fernando Monteiro, Brad Amorati, Yenling Yeh, Simon Roger Poulson, Antonio Pinheiro Faciola

**Affiliations:** 1 Department of Agriculture, Nutrition, and Veterinary Sciences, University of Nevada, Reno, Nevada, United States of America; 2 Department of Animal Sciences, Federal University of Viçosa, Viçosa, Minas Gerais, Brazil; 3 Department of Animal Sciences, Maringá State University, Maringá, Paraná, Brazil; 4 Department of Geological Sciences & Engineering, University of Nevada, Reno, Nevada, United States of America; National Institute of Agronomic Research, FRANCE

## Abstract

The objective of this study was to evaluate the effects of partially replacing dry ground corn with glycerin on ruminal fermentation using a dual-flow continuous culture system. Six fermenters (1,223 ± 21 ml) were used in a replicated 3x3 Latin square arrangement with three periods of 10 d each, with 7 d for diet adaptation and 3 d for sample collections. All diets contained 75% concentrate and three dietary glycerin levels (0, 15, and 30% on dry matter basis), totaling six replicates per treatment. Fermenters were fed 72 g of dry matter/d equally divided in two meals/d, at 0800 and 2000 h. Solid and liquid dilution rates were adjusted daily to 5.5 and 11%/h, respectively. On d 8, 9, and 10, samples of 500 ml of solid and liquid digesta effluent were mixed, homogenized, and stored at -20°C. Subsamples of 10 ml were collected and preserved with 0.2 mL of a 50% H_2_SO_4_ solution for later determination of NH_3_-N and volatile fatty acids. Microbial biomass was isolated from fermenters for chemical analysis at the end of each experimental period. Data were analyzed using the MIXED procedure in SAS with α = 0.05. Glycerin levels did not affect apparent digestibility of DM (*P*_*Lin*._ = 0.13; *P*_*Quad*._ = 0.40), OM (*P*_*Lin*._ = 0.72; *P*_*Quad*._ = 0.15), NDF (*P*_*Lin*._ = 0.38; *P*_*Quad*._ = 0.50) and ADF (*P*_*Lin*._ = 0.91; *P*_*Quad*._ = 0.18). Also, glycerin inclusion did not affect true digestibility of DM (*P*_*Lin*._ = 0.35; *P*_*Quad*._ = 0.48), and OM (*P*_*Lin*._ = 0.08; *P*_*Quad*._ = 0.19). Concentrations of propionate (*P* < 0.01) and total volatile fatty acids (*P* < 0.01) increased linearly and concentrations of acetate (*P* < 0.01), butyrate (*P* = 0.01), *iso*-valerate (*P* < 0.01), and total branched-chain volatile fatty acids, as well as the acetate: propionate ratio (*P* < 0.01) decreased with glycerin inclusion. Linear increases on NH_3_-N concentration in digesta effluent (*P* < 0.01) and on NH_3_-N flow (*P* < 0.01) were observed due to glycerin inclusion in the diets. Crude protein digestibility (*P* = 0.04) and microbial N flow (*P* = 0.04) were greater in the control treatment compared with the other treatments and responded quadratically with glycerin inclusion. Furthermore, the inclusion of glycerin linearly decreased (*P* = 0.02) non-ammonia N flow. Glycerin levels did not affect the flows of total N (*P*_*Lin*._ = 0.79; *P*_*Quad*._ = 0.35), and dietary N (*P*_*Lin*._ = 0.99; *P*_*Quad*._ = 0.07), as well as microbial efficiency (*P*_*Lin*._ = 0.09; *P*_*Quad*._ = 0.07). These results suggest that partially replacing dry ground corn with glycerin may change ruminal fermentation, by increasing total volatile fatty acids, and propionate concentration without affecting microbial efficiency, which may improve glucogenic potential of beef cattle diets.

## Introduction

Increased demands for corn in recent years due to ethanol production and human utilization have contributed to increased corn prices. Because corn has been traditionally used as the main energy source in cattle diets, alternative energy sources are needed. Glycerin is an important by-product of biodiesel production and can be converted to propionate in the rumen [1], which is the main glucogenic precursor in ruminants [2]. Glucose is the main carbon source used for fatty acid synthesis [3], which may lead to an increase in marbling of meat [4]. Furthermore, glycerin may also increase starch digestibility when included in diets for beef cattle [1], which could increase total volatile fatty acid (**VFA**) concentration and improve beef cattle energy efficiency. Therefore, partial replacement of corn with an ethanol by-product such as glycerin may improve energy efficiency in beef cattle, which is desirable from both energetic and environmental perspectives.

Studies have reported the effects of glycerin inclusion on ruminal fermentation when glycerin was included at up to 20% [5,6] in beef cattle diets; however, the effects of higher inclusion levels in high concentrate diets for finishing cattle have not been evaluated and the maximum levels of glycerin use have not been established. The objective of this study was to evaluate the effects of partially replacing dry ground corn with glycerin on ruminal parameters using a dual-flow continuous culture system. We hypothesized that glycerin may partially replace corn as an energy source in finishing beef cattle diets and may be included at concentrations up to 30% [dry matter (**DM**) basis], increasing propionate concentration without compromising ruminal fermentation, digestibility, microbial N flow and microbial efficiency in a dual-flow continuous culture system.

## Materials and Methods

### Ethical approval

The care and handling of all experimental animals, including ruminal cannulation were conducted under protocols approved by the University of Nevada, Reno Institutional Animal Care and Use Committee (IACUC protocol number 00588). Ruminal cannulations were conducted 12 months prior to the experiment by Dr. Walter Mandeville, a clinical veterinarian from the University of Nevada, Reno. Standard surgical preparation was used on the left paralumbar fossa and sterile drapes and gowns were utilized, hair on the surgery area was clipped using a #40 blade and the area was scrubbed three times alternating povidone-iodine and 70% alcohol. Local anesthesia was provided using an inverted L block with 150 cc 2% lidocaine hydrochloride and butorphanol tartrate was administered intravenously at a dosage of 0.05 mg/kg. Animals were restrained in a stanchion and a 10 cm incision was made through the skin and subcutaneous tissue and the peritoneum was incised to open the abdominal cavity. Stay sutures were placed in an avascular area of the rumen and the rumen was sutured to the peritoneum and the skin. A section of the rumen was excised and a ruminal cannula (7.5 cm in diameter, Bar Diamond Inc., Parma, ID) was inserted into the rumen fistula. To control post-operative infection, 30 cc penicillin was given once a day for seven days and post-operative discomfort was alleviated by administering flunixin meglumine at dosage of 1.6 mg/kg intravenously once daily for seven days.

### Experiment Design and Diets

Six 1,223 ± 21 ml dual-flow continuous-culture fermenters, similar to that described by Hoover *et al*. [7], were used in three consecutive 10 d periods with 7 d for adaptation and 3 d for sampling. The experimental design included two 3 x 3 Latin squares, which were run simultaneously with six replicates per treatment. The treatments were: inclusion of 0, 15, and 30% (DM basis) of glycerin (99.7% of purity; Nature’s Oil, Streetsboro, OH, USA) replacing corn in finishing beef diets. Experimental diets were composed of 25% wheat straw and 75% concentrate (DM basis) and were formulated to meet the nutrient requirements of beef cattle recommendations [8]. Dietary ingredients were ground to pass a 2 mm screen (Wiley mill; Thomson Scientific Inc., Philadelphia, PA) and diets were formulated to be isonitrogenous and urea was used to equalize nitrogen levels. Ingredient proportion and chemical composition of the experimental diets are presented in Table 1.

**Table 1:** Ingredient and chemical composition of experimental diets.

Item^1^	Glycerin, %		
	0	15	30
Ingredient, % DM			
Wheat straw	25.0	25.0	25.0
Dry ground corn	53.3	37.9	22.4
Glycerin^2^	0.0	15.0	30.0
Soybean meal	18.7	18.7	18.7
Urea	0.00	0.44	0.89
Mineralized salt^3^	3.00	3.00	3.00
Composition, % DM			
DM, %	88.9	90.0	91.1
OM	92.7	93.0	93.2
NDF	25.6	23.7	22.2
CP	14.5	14.5	14.5
Ether extract	3.43	2.67	1.91

^1^DM = dry matter; OM = organic matter; NDF = neutral detergent fiber; CP = crude protein.

^2^Purity of 99.7% (Nature’s Oil, Streetsboro, OH, USA)

^3^Provided (per kg of DM): 955 g of NaCl, 3,500 ppm of Zn, 2,000 ppm of Fe, 1,800 ppm of Mn, 280 ppm of Cu, 100 ppm of I, and 60 ppm of Co.

### Dual-flow Continuous Culture System Operation and Sample Collection

On d 1, fermenters were inoculated with rumen fluid from two rumen cannulated Aberdeen Angus steers (Average BW of 770 kg). Steers were maintained on a total mix diet of 40% grass hay, 49% dry ground corn, 8% soybean meal, and 3% mineralized salt. Two hours after feeding, 10 L of rumen contents were collected, immediately filtered through 4 layers of cheesecloth and placed into pre-warmed thermal containers. Equal amounts of rumen content from each animal were homogenized thoroughly by agitation, infused with N_2_ to maintain the anaerobic environment and adjusted to 39°C by submerging a 5,000 mL Erlenmeyer flask in a pre-heated water bath. The rumen fluid was poured into each of the pre-warmed fermenters until it cleared the overflow spout.

In accordance with Hoover *et al*. [7], fermentation conditions were maintained constant with a temperature of 39°C and N_2_ (40 ml/min) was infused into the fermenters to maintain the anaerobic conditions of the system. However, the pH was not controlled and urea was added to the artificial saliva to simulate recycled N [9]. Individual Cole-Parmer pH controllers (Model 5997–20) were used to monitor the pH of each fermenter. A central propeller apparatus driven by magnets was used to continuously agitate the fermenters contents. Artificial saliva infusion and filtered liquid flows were set to maintain solids and liquids rates at 5.5 and 11%/h, respectively, by adjusting buffer input and solid and liquid removal [10], to mimic real passage rates in beef cattle [11].

Fermenters were fed 72 g of DM/d of diet equally divided in two meals per d, at 0800 and 2000 h. Digesta effluent (solid and liquid) were collected in 4 L plastic containers. At 0730 h of each day of the adaptation period, the containers were weighed and contents were discarded. Twenty-four hours prior to the first collection and during the 3 d sampling period (d 8, 9, and 10), each container received 20 mL of 50% H_2_SO_4_ and were immersed halfway in a chilled (2–4°C) water bath to stop microbial and enzymatic activities. On d 8, 9, and 10, samples of 500 ml of solid and liquid digesta effluent were mixed, homogenized (T25 basics, IKA Works, Inc, Wilmington, NC 28405), and stored at -20°C. A subsample of 10 ml was filtered through two layers of cheesecloth. Then, 0.2 mL of a 50% H_2_SO_4_ solution was added for later determination of ammonia nitrogen (**NH**_**3**_ **-N**) [12] and VFA. At the end of each period, digesta effluent from the three sampling days were composited by fermenter and freeze dried for further analysis.

On d 5, digesta effluent (solid and liquid) was homogenized by vigorous hand shaking and samples were collected to determine the digesta effluent background ^15^N abundance [13]. Then, 0.077 g of 10.2% excess ^15^(NH_4_)_2_SO_4_ (Sigma-Aldrich Co., St. Louis, MO) was infused into each fermenter to instantaneously label the NH_3_-N Pool. Saliva was reformulated and 0.077 g/L of enriched ^15^(NH_4_)_2_SO_4_ (Sigma-Aldrich Co., St. Louis, MO) were added in replacement of isonitrogenous amounts of urea to maintain a steady-state concentration of ^15^N enrichment in fermenters.

Ruminal liquid from fermenters were sampled on d 8 to determine NH_3_-N concentration in different time-points; 10 mL of liquid samples were collected at different time points: 0, 1, 2, 4, 6, 8, and 10 h after feeding, filtered through a double layer of cheesecloth. Then, the samples were analyzed for NH_3_-N [12]. At the same time-points, pH was measured with an Accumet portable AP61 pH meter (Fisher Scientific, Atlanta, GA). At the end of each 10 d experimental period, the fermenter contents were strained through 2 layers of cheesecloth, and centrifuged at 1,000 x g for 10 min to remove undigested substrate and possibly some attached microbial biomass [14]. Then, the supernatant was centrifuged at 10,000 x g for 20 min to isolate pure microbial biomass pellets [14]. Supernatant was discarded and microbial pellets were freeze dried and stored for further ^15^N enrichment analysis [13].

### Chemical Analyses and Calculations

Feed and digesta effluent samples were analyzed for DM (method 934.01), ash (method 938.08), crude protein (**CP**; method 984.13), and ether extract (method 920.85) according to AOAC [15]. The organic matter (**OM**) was calculated as the difference between DM and ash contents. For neutral detergent fiber (**NDF**) and acid detergent fiber (**ADF**), samples were sequentially analyzed, treated with alpha thermo-stable amylase without sodium sulfite according to Van Soest *et al*. [16] and adapted for the Ankom200 Fiber Analyzer (Ankom Technology, Macedon, NY). Samples of microbial pellet and digesta effluent background were analyzed for DM, CP, and ash as detailed previously for feed samples.

Volatile fatty acid concentrations in the digesta effluent were determined using gas chromatography (Varian Model 3800; Varian, Inc, Walnut Creek, CA; equipped with a glass column [180 cm x 4 mm i.d.]) packed with GP 10% SP-1200/1% H_3_PO_4_ on 80/100 Chromosorb WAW [Supelco, Bellefonte, PA]), and N was used as a carrier gas at a flow rate of 85 mL/min^-1^. The NH_3_-N concentrations (on fermenter and effluent digesta) were determined by colorimetric as described by Chaney and Marbach [12].

Background, digesta effluent and microbial pellets samples were analyzed for total N and ^15^N enrichment according to Werner et al. [17]. Isotope analyses were performed using an Eurovector model 3028 elemental analyzer interfaced to a Micromass Isoprime stable isotope ratio mass spectrometer. Microbial N flow and microbial efficiency were calculated as follows: Microbial N flow (expressed in g/d) = [non-ammonia N (**NAN**) flow x percentage of ^15^N atom excess of digesta effluent]/(percentage of ^15^N atom of microbial pellet), with ^15^N digesta effluent background subtracted from ^15^N enrichment. Microbial efficiency = Microbial N flow (g) / OM truly digestible (kg) [13]. According to Soder et al. [18], apparent and true digestibilities were calculated as follows (using DM as an example): DM apparently digested (%) = [(g of DM intake—g of effluent flow DM) / g of DM intake] x 100; DM truly digested (%) = {[g of DM intake—(g of effluent flow DM—g of microbial DM)] / g of DM intake} x 100. Effluent was corrected for grams of buffer in both equations.

### Statistical Analysis

All results were tested for normality [19], and they followed normal distribution (*P* > 0.05). All statistical procedures were carried out using SAS 9.2 for Windows (Statistical Analysis System Institute, Inc., Cary, NC, USA) with α = 0.05. Variance Components was used as the covariance structure in the two models used. Nutrient flow and digestibility, VFA, N metabolism (including NH_3_-N from digesta effluent), and microbial efficiency were analyzed for linear and quadratic responses using the following model:

$${\text{Y}}_{\text{ijk}}{\text{ = B}}_{\text{0}}{\text{ + B}}_{\text{1}}{\text{X}}_{\text{i}}{\text{ + B}}_{\text{2}}{\text{X}}_{\text{i}}^{\text{2}}{\text{ + P}}_{\text{j}}{\text{ + A}}_{\text{k}}{\text{ + e}}_{\text{ijk}},$$

where:

Y_ijk_ is the observed measurement of the i^th^ level of glycerin inclusion in the diet of the j^th^ period, and of the k^th^ experimental unit; i = 1, 2, 3 (levels of inclusion of glycerin as a replacement of corn), B_0_, B_1_, B_2_ = regression parameters of the model; X_i_ = effect of i^th^ level of fixed quantitative factor (replacement of glycerin with corn); P_j_ = effect of level of random factor period; A_k_ = effect of level of random factor fermenter; e_ijkl_ = residual error, assuming e_ijkl_ ~ N (0, s^2^).

Only pH and NH_3_-N data collected from fermenters over time were analyzed as repeated measurements and the subject of the repeated statement was the fermenter within period. All one-way, two-way and three-way interactions were tested. Outliers were identified when the Studentized residue was greater than 2.5 or smaller than -2.5. All non-significant effects (*P* > 0.05) were removed from the model to determine the final equation. Therefore, pH and NH_3_-N were analyzed for linear, quadratic, and cubic responses of time using the following model:

$${\text{Y}}_{\text{ijklm}}{\text{ = B}}_{\text{0}}{\text{ + B}}_{\text{1}}{\text{X}}_{\text{i}}{\text{ + B}}_{\text{2}}{\text{X}}_{\text{i}}^{\text{2}}{\text{ + B}}_{\text{3}}{\text{T}}_{\text{k}}{\text{ + B}}_{\text{4}}{\text{X}}_{\text{i}}{\text{T}}_{\text{k}}{\text{ + B}}_{\text{5}}{\text{X}}_{\text{i}}^{\text{2}}{\text{T}}_{\text{k}}{\text{ + B}}_{\text{6}}{\text{T}}_{\text{k}}^{\text{2}}{\text{ + B}}_{\text{7}}{\text{X}}_{\text{i}}{\text{T}}_{\text{k}}^{\text{2}}{\text{ + B}}_{\text{8}}{\text{X}}_{\text{i}}^{\text{2}}{\text{T}}_{\text{k}}^{\text{2}}{\text{ + B}}_{\text{9}}{\text{T}}_{\text{k}}^{\text{3}}{\text{ + B}}_{\text{10}}{\text{X}}_{\text{i}}{\text{T}}_{\text{k}}^{\text{3}}{\text{ + B}}_{\text{11}}{\text{X}}_{\text{i}}^{\text{2}}{\text{T}}_{\text{k}}^{\text{3}}{\text{ + P}}_{\text{j}}{\text{ + A}}_{\text{l}}{\text{+e}}_{\text{ijklm}}\text{,}$$

where:

Y_ijklm_ is the observed measurement of the i^th^ level of glycerin inclusion in the diet of the j^th^ period, of the k^th^ time, of the m^th^ experimental unit replication; i = 1, 2, 3 (levels of inclusion of glycerin as a replacement of corn), B_0_, B_1_, … B_11_ = regression parameters of the model; X_i_ = effect of i^th^ level of fixed quantitative factor (replacement of glycerin with corn); T_k_ = effect of k^th^ level of time; P_j_ = effect of level of random factor period; A_l_ = effect of level of random factor fermenter; e_ijklm_ = residual error, assuming e_ijklm_ ~ N (0, s^2^).

## Results

### Apparent and True Digestibility

Glycerin levels did not affect apparent digestibility of DM (*P*_*Lin*._ = 0.13; *P*_*Quad*._ = 0.40), OM (*P*_*Lin*._ = 0.72; *P*_*Quad*._ = 0.15), NDF (*P*_*Lin*._ = 0.38; *P*_*Quad*._ = 0.50) and ADF (*P*_*Lin*._ = 0.91; *P*_*Quad*._ = 0.18), which averaged 33.5 ± 2.63, 41.6 ± 2.56, 75.6 ± 4.07, and 70.6 ± 3.17%, respectively (Table 2). Also, glycerin inclusion did not affect true digestibility of DM (*P*_*Lin*._ = 0.35; *P*_*Quad*._ = 0.48), and OM (*P*_*Lin*._ = 0.08; *P*_*Quad*._ = 0.19), which averaged 57.7 ± 6.00%, and 59.6 ± 3.92%, respectively.

**Table 2:** Effect of glycerin inclusion on apparent and true digestibility of dietary nutrients in dual-flow continuous culture system.

Item^1^	Glycerin, %			SEM	*P*-value	
	0	15	30		Linear	Quadratic
Apparent Digestibility, %						
DM	32.7	35.0	35.1	0.64	0.13	0.40
OM	40.9	42.7	41.3	0.60	0.72	0.15
NDF	76.1	76.5	74.3	0.96	0.38	0.50
ADF	70.0	72.1	69.7	0.75	0.91	0.18
True Digestibility, %						
DM	60.1	56.3	56.8	1.42	0.35	0.48
OM	60.8	57.0	57.3	0.92	0.08	0.19

^1^DM = dry matter; OM = organic matter; NDF = neutral detergent fiber; ADF = acid detergent fiber.

### Volatile fatty acids

The inclusion of glycerin linearly increased total VFA (*P* < 0.01) and propionate concentrations (*P* < 0.01; Table 3). Compared to the control (0% glycerin), total VFA and propionate concentrations increased 11 and 105%, respectively, when 30% glycerin was fed. Concentrations of acetate (*P* < 0.01), butyrate (*P* = 0.01), *iso*-valerate (*P* < 0.01), and total BCVFA (*P* < 0.01), as well as the acetate: propionate ratio (*P* < 0.01) decreased linearly as glycerin replaced corn. *Iso*-butyrate concentration was greater for control compared with the other treatments and responded quadratically (*P* = 0.03) as dietary glycerin increased. Glycerin levels did not affect valerate concentration (*P*_*Lin*._ = 0.45; *P*_*Quad*._ = 0.53), which averaged 1.06 ± 0.21%.

**Table 3:** Effect of glycerin inclusion on total and individual VFA concentrations in dual-flow continuous culture system.

Item^1^	Glycerin, %			SEM	*P*-value	
	0	15	30		Linear	Quadratic
Total VFA, mM	113	123	125	1.81	< 0.01	0.12
VFA, mM						
Acetate	66.5	59.9	48.8	2.17	< 0.01	0.42
Propionate	25.3	43.5	58.6	3.55	< 0.01	0.47
Butyrate	17.7	16.4	13.3	0.82	0.01	0.52
Valerate	1.33	1.18	1.21	0.07	0.45	0.53
*Iso*-Butyrate	0.42	0.24	0.22	0.03	< 0.01	0.03
*Iso*-Valerate	1.61	1.19	0.69	0.11	< 0.01	0.74
BCVFA, mM	2.98	2.62	2.03	0.31	< 0.01	0.07
Acetate: propionate	2.65	1.41	0.84	0.19	< 0.01	0.22

^1^VFA = volatile fatty acids; BCVFA = Branched-chain VFA.

### Ruminal pH and NH_3_-N over Time

**Fig 1:**
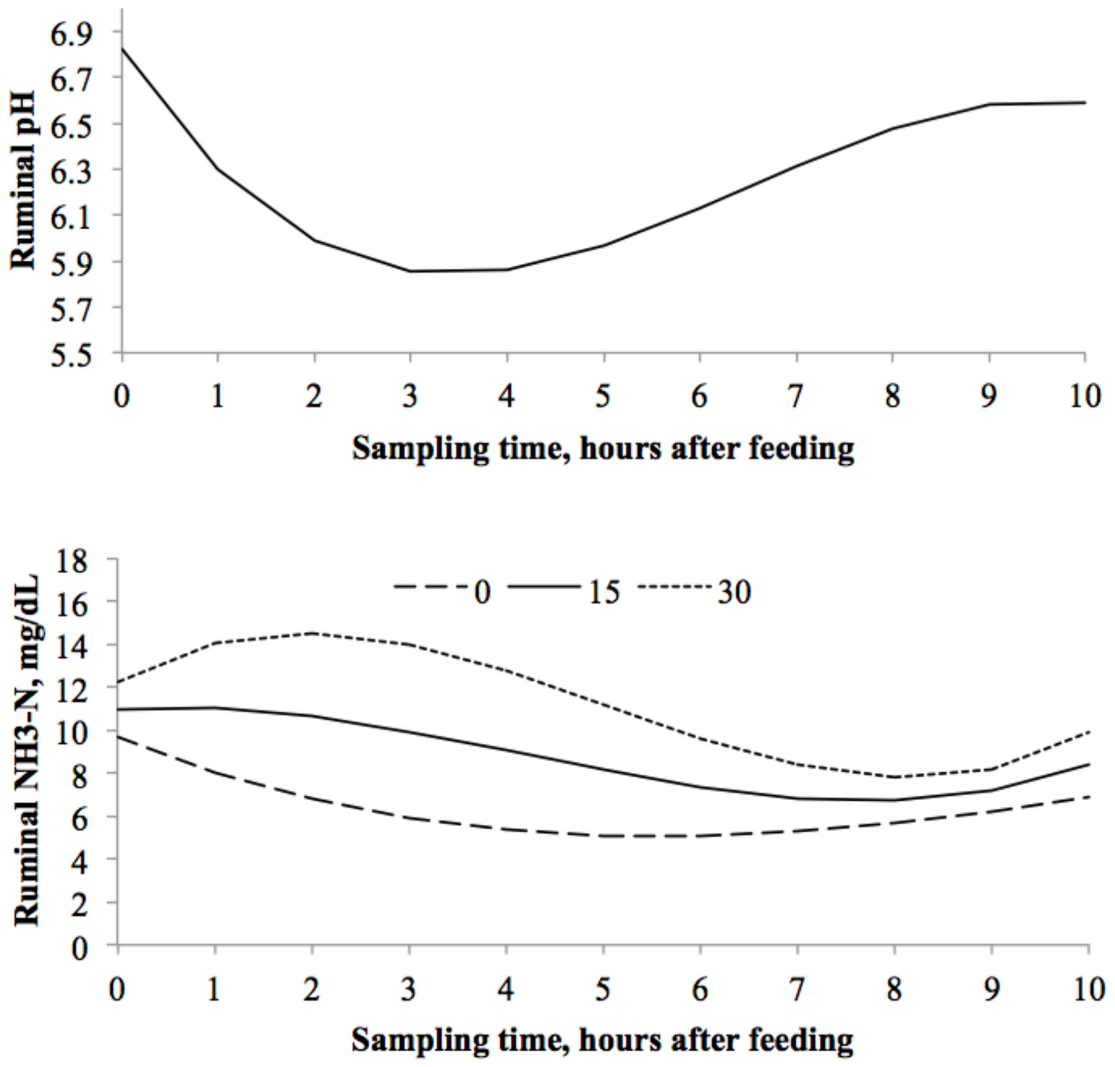
Effect of glycerin inclusion on ruminal pH and NH_3_-N over time in dual-flow continuous culture system. pH = 6.825 ± 0.1727 - (TIME x 0.6487 ± 0.07149) + (TIME^2^ x 0.1281± 0.01751)—(TIME^3^ x 0.00656 ± 0.001143); R^2^ = 0.999, MSE = 0.139. NH_3_-N = 9.6399 ± 1.4148 + (glycerin x 0.08728 ± 0.05779) + (glycerin x TIME x 0.146 ± 0.06041)—(TIME x 1.8245 ± 1.1698)—(glycerin x TIME^2^ x 0.03459 ± 0.0148) + (TIME^2^ x 0.2095 ± 0.2866) + (glycerin x TIME^3^ x 0.002012 ± 0.000966)—(TIME^3^ x 0.00546 ± 0.01871); R^2^ = 0.381, MSE = 11.75.

Two equations were generated to describe ruminal pH and NH_3_-N over time (Fig 1). No differences were observed (*P* = 0.09) on pH among different levels of glycerin. However, sampling time had a quadratic effect (*P* < 0.01) on ruminal pH, with the critical point (5.85) being reached 3 h after feeding. There was an interaction between inclusion of glycerin in the diet and sampling time for ruminal NH_3_-N (*P* = 0.02). Sampling time had a cubic effect (*P* = 0.04) on concentrations of ruminal NH_3_-N, which were higher throughout the day for fermenters fed 30% glycerin.

### Nitrogen Metabolism and Microbial Efficiency

A linear increase of NH_3_-N concentration in digesta effluent (*P* < 0.01), and NH_3_-N flow (*P* < 0.01) were observed due to inclusion of glycerin in the diets (Table 4). The digesta effluent NH_3_-N concentration of 30% glycerin inclusion was about 61% greater than the control treatment. The CP digestibility (*P* = 0.04) and microbial N flow (*P* = 0.04) were greater for control (0% glycerin) compared to the other treatments and responded quadratically as glycerin replaced corn. Furthermore, the inclusion of glycerin linearly decreased NAN flow (*P* = 0.02). Glycerin levels did not affect the flows of total N (*P*_*Lin*._ = 0.79; *P*_*Quad*._ = 0.35), and dietary N (*P*_*Lin*._ = 0.99; *P*_*Quad*._ = 0.07), as well as microbial efficiency (*P*_*Lin*._ = 0.09; *P*_*Quad*._ = 0.07), which averaged 1.53 ± 0.14 g/d, 0.23 ± 0.11 g/d, and 22.3 ± 3.23 g of microbial N/kg of OM truly digested, respectively.

**Table 4:** Effect of glycerin inclusion on nitrogen metabolism of rumen microorganisms in dual-flow continuous culture system.

Item^1^	Glycerin, %			SEM	*P*-value	
	0	15	30		Linear	Quadratic
NH_3_-N, mg/100ml	11.0	13.1	17.8	0.90	< 0.01	0.24
CP digestibility, %	79.4	67.6	65.9	1.82	< 0.01	0.04
Nitrogen flow, g/d						
Total N	1.57	1.49	1.54	0.03	0.79	0.35
NH_3_-N	0.35	0.41	0.56	0.03	< 0.01	0.22
NAN	1.24	1.11	0.98	0.05	0.02	0.95
Microbial N	0.99	0.75	0.79	0.04	0.01	0.04
Dietary N	0.20	0.30	0.20	0.03	0.99	0.07
Microbial efficiency^2^	23.9	19.4	20.4	0.78	0.09	0.07

^1^NH_3_-N = ammonia nitrogen; CP = crude protein; NAN = non-ammonia nitrogen; OM = organic matter.

^2^Microbial efficiency = g of microbial N/kg of OM truly digested.

## Discussion

### Apparent and True Digestibility

We hypothesized that glycerin could replace corn and included at up to 30% in beef cattle diets without compromising apparent and true digestibility in a dual-flow continuous culture system. As expected, the replacement of corn with glycerin did not change any digestibility parameter. Lack of effects in ruminal DM and OM digestibility has been observed when glycerin was included in cattle diets, both *in vitro* [20] and *in vivo* [6,21]. These authors suggest that quick microbial adaptation and fast glycerin ruminal turnover [22,23,24] as well as high VFA production [23,25,26] may be possible reasons for this observation. According to Van Soest [27], the rates of digestion and passage are the two main factors that affect the digestibility. As the passage rate was set to be the same for all treatments, it seems that microbial adaptation played a role at maintaining adequate nutrient digestion among treatments. The increase in VFA concentration and the lack of differences for microbial efficiency observed in this study help explaining the results observed for digestibility. Therefore, these results suggest that glycerin inclusion at up to 30% in high concentrate diets has no detrimental effects on ruminal digestibility.

### Volatile fatty acids

The findings in this study contradict our hypothesis that glycerin inclusion would not modify total VFA concentration in the rumen. The increase on total VFA concentration is probably related to the fact that glycerin has an additive relationship with corn-starch digestion [1]. Hales *et* al. [1] observed increase on starch digestibility when glycerin was included in diets of beef steers. According to these authors, glycerin stimulates amylolitic bacteria, such as *Selenomonas ruminantium* [28] and *Streptococcus bovis* [29], which makes glycerin an ingredient with potential to increase energy availability in finishing diets, which are rich in starch.

Also, increases in total VFA concentration might be related to higher ruminal NH_3_-N concentration [30]. Other studies have found increases in VFA concentration due to higher NH_3_-N levels even when NH_3_-N levels in the control diets were not limiting [31,32,33]. Thus, higher ruminal NH_3_-N observed in glycerin treatments may also explain the increase on total VFA concentration for these treatments in this study. Results for total VFA have been conflicting with previous studies that observed no effects [5,34,35,36,37] or an increase [22,23,24,38] in total VFA concentration in the rumen when glycerin was fed.

The shift on VFA profile, with an increase in propionate concentration at the expense of acetate concentration and the decrease on acetate: propionate ratio were expected. However, the increase in propionate was also associated with a decrease in butyrate and *iso*-acids concentrations. Glycerin is preferentially fermented to propionate rather than acetate [5,20]. As mentioned before, glycerin stimulates *Selenomonas ruminantium* subsp. *lactilyca* [28], which are propionate producing bacteria [29]. Moreover, one of the main glycerin-fermenting bacteria, *Anaerovibrio lipolytica* does not have acetate as the main fermentation product when glycerin is the substrate [29]. Similar results were observed by Avila et al. [34] when the shift on VFA profile occurred, with increase of propionate at the expense of acetate and butyrate concentrations in an *in vitro* experiment when glycerin (99.5% purity) was included at up to 21% (DM basis). Rico et al. [35] also noted an increase in propionate and a decrease in acetate concentrations when fermenters in a continuous culture system were fed diets containing up to 8% glycerin (DM basis). Moreover, Shin et al. [36] observed an increase in propionate molar proportions at the expense of acetate in rumen cannulated Holstein cows when glycerin (75.8% purity) was included in the diet at up to 10% of DM. Furthermore, Avila-Stagno et al. [38] observed increases in total VFA and propionate concentrations and decrease in acetate: propionate ratio in a semi-continuous culture system when glycerin (99.5% purity) was included at up to 15% (DM basis) in forage-based diets. Other studies indicated that dietary glycerin decreases acetate: propionate ratio when included at up to 10% [1], 17.5% [21] and 20% [5] in beef cattle diets. The reduction in *iso*-acids in treatments with glycerin may be explained by the decrease on CP digestibility that was observed in this study. *Iso*-acids are produced mainly by the amino acid fermentation in the rumen [39]. Nevertheless, the increase in propionate concentrations in this study indicates higher glucogenic potential when glycerin replaces corn in beef cattle diets. This might be important for finishing animals since glucose is the main precursor of intramuscular fatty acid synthesis [40].

### Ruminal pH and NH3-N over Time

Partial dietary replacement of corn with glycerin did not affect ruminal pH over time in this study. As no variation was observed in digestibility parameters and microbial efficiency, the lack of effect on pH is justified. Ruminal pH is associated to ruminal VFA fermentation [41]. Ruminal pH had similar pattern for all treatments, reaching the critical point (peak of minimum values) 3 h after feeding. The glycerin has a fast fermentation in the rumen [22,23,24], such that 50 to 80% of glycerin disappears within 4 h [42], which could decrease ruminal pH faster than corn. However, corn was ground in this study, allowing faster access to starch-fermenting bacteria, which might have caused similar fermentation rate than glycerin. Similar results were observed by Rico et al. [35], when glycerin inclusion did not affected pH in a continuous culture experiment in which diet components were ground through a 4-mm mesh screen. Moreover, pH values observed in this study were within the optimal recommended values for cattle fed high concentrate diets [43,44]. However, the rumen might be more buffered in this study than in *in vivo* situations, since continuous culture system utilizes artificial saliva with constant rate of infusion.

The higher ruminal NH_3_-N concentration in fermenters fed diets with glycerin are likely related to the higher dietary urea levels in the glycerin diets. Urea contributed with 0, 8.6, and 17.3% of the total dietary CP for the treatments with 0, 15, and 30% glycerin; respectively. Urea is quickly converted to NH_3_-N in the rumen, which can increase NH_3_-N levels faster than its use by microorganisms [44]. A fast release of nitrogen may cause an asynchrony of NH_3_-N and energy in the ruminal environment. Thus, the low ruminal NH_3_-N concentration observed in the control treatment (0% glycerin) in this study may be related to a better nitrogen: energy synchrony.

### Nitrogen Metabolism and Microbial Efficiency

We hypothesized that glycerin inclusion would not modify nitrogen metabolism in a dual-flow continuous culture system. However, compared to corn, glycerin increased NH_3_-N flow and decreased CP digestibility, and the flows of NAN and microbial-N. Nevertheless, there were no differences among treatments for total N flow and microbial efficiency. As mentioned before, glycerin increased NH_3_-N concentration in the rumen, likely due to dietary urea. Thus, the rapid accumulation of ruminal NH_3_-N could not be matched by the microorganism’s rate of N incorporation. This may have reduced microbial N flow, and CP digestion in glycerin diets. Wang *et al*. [24] also observed that the *in situ* ruminal CP digestibility was reduced with increasing doses of glycerin in beef steers diets. However, in the same study it was observed an increase in total tract DM, OM, NDF, and CP digestibilities. The authors concluded that glycerin inclusion improved post ruminal nutrient digestibility. Furthermore, Hales *et al*. [1] observed a linear increase trend in post ruminal N digestibility when glycerin was included at up to 10% in diets of beef steers. Furthermore, in our study the lower microbial N flow in fermenters fed glycerin was not capable of affecting microbial efficiency, which did not differ among treatments. Ramos and Kerley [5] observed a linear increase in microbial efficiency in fermenters fed diets with glycerin at up to 20% of DM. According to these authors, this increase in microbial efficiency occurred mainly due to the decrease on OM digestibility found in their study. In the current study, glycerin inclusion did not affect ruminal OM digestibility as well as microbial efficiency. Therefore, these results lead us to conclude that glycerin inclusion in beef cattle diets may change N flow partitioning, reducing microbial-N, despite not changing total dietary nitrogen flow as well as microbial efficiency in a dual-flow continuous culture system.

## Conclusions

These results suggest that partially replacing corn with glycerin may change ruminal fermentation; increasing total VFA, and propionate concentration without affecting rumen microbial efficiency in a dual-flow continuous culture system. From a glucogenic perspective, these findings suggest that the inclusion of glycerin would increase dietary glucogenic potential, which is especially important for marbling in finishing animals. However, glycerin inclusion caused a decrease in microbial N flow, which might or might not be desirable depending on the animals’ requirements. Therefore, under these experimental conditions, glycerin inclusion increased glucogenic precursors and effectively replaced dietary corn at up to 30% of the diet.

## Supporting Information

S1 Table Raw Data. (XLSX)

## Abbreviations

ADF: acid detergent fiber
BCVFA: branched-chain volatile fatty acids
CP: crude protein
DM: dry matter
NAN: non-ammonia nitrogen
NH_3_-N: ammonia nitrogen
NDF: neutral detergent fiber
OM: organic matter
VFA: volatile fatty acids
